# The Multivesicular Body and Autophagosome Pathways in Plants

**DOI:** 10.3389/fpls.2018.01837

**Published:** 2018-12-12

**Authors:** Yong Cui, Yilin He, Wenhan Cao, Jiayang Gao, Liwen Jiang

**Affiliations:** ^1^Centre for Cell and Developmental Biology, School of Life Sciences, The Chinese University of Hong Kong, Shatin, Hong Kong; ^2^State Key Laboratory of Agrobiotechnology, The Chinese University of Hong Kong, Shatin, Hong Kong; ^3^The Chinese University of Hong Kong Shenzhen Research Institute, Shenzhen, China

**Keywords:** MVBs, autophagosomes, vacuolar degradation, crosstalk, protein structure, conserved domains

## Abstract

In eukaryotic cells, the endomembrane system consists of multiple membrane-bound organelles, which play essential roles in the precise transportation of various cargo proteins. In plant cells, vacuoles are regarded as the terminus of catabolic pathways whereas the selection and transport of vacuolar cargoes are mainly mediated by two types of organelles, multivesicular bodies (MVBs) also termed prevacuolar compartments (PVCs) and autophagosomes. MVBs are single-membrane bound organelles with intraluminal vesicles and mediate the transport between the *trans-*Golgi network (TGN) and vacuoles, while autophagosomes are double-membrane bound organelles, which mediate cargo delivery to the vacuole for degradation and recycling during autophagy. Great progress has been achieved recently in identification and characterization of the conserved and plant-unique regulators involved in the MVB and autophagosome pathways. In this review, we present an update on the current knowledge of these key regulators and pay special attention to their conserved protein domains. In addition, we discuss the possible interplay between the MVB and autophagosome pathways in regulating vacuolar degradation in plants.

## Introduction

**FIGURE 1:**
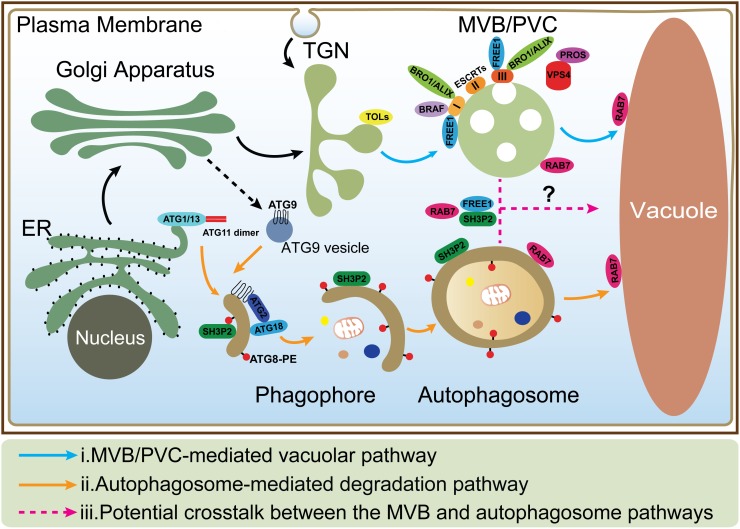
The MVB and autophagosome pathways in plant cells. (i) In MVB/PVC-mediated vacuolar pathway, proteins with vacuolar sorting signal are sorted into MVBs/PVCs and then deposited in the vacuole for degradation; (ii) In autophagic pathway, autophagosomes derive from ER and deliver cargoes into the vacuole for degradation and recycling. ATG9 vesicle is essential for ER-derived autophagosome formation; (iii) The possible crosstalk between the MVB and autophagosome pathways (as indicated by question mark) is also depicted by the dashed lines. MVB, multivesicular body; PVC, prevacuolar compartment; ER, endoplasmic reticulum; TGN, trans-Golgi network.

Vacuoles are the major sites for both storage and metabolism in plant cells and play essential roles during plant growth and development (Shimada et al., 2018). Plant vacuoles are generally classified into protein storage and lytic vacuoles based on their distinct functions (Eisenach et al., 2015). Protein storage vacuoles serve as the main repository of protein in seeds, while lytic vacuoles act as the primary catabolic compartment in vegetative cells, as they contain hydrolytic enzymes that can break down various biomolecules for recycling. Before degradation inside lytic vacuoles, cargo proteins are first sequestered into certain types of organelles such as multivesicular bodies (MVBs) or prevacuolar compartments (PVCs), and autophagosomes (Zhuang et al., 2013; Cui et al., 2016; Marshall and Vierstra, 2018). Under normal conditions, proteins, such as hydrolytic enzymes and membrane receptors, are continuously transported to the vacuoles via single-membrane bound MVBs. The selection of soluble and membrane cargoes is mediated by vacuolar sorting receptors (VSRs) and the endosomal sorting complexes required for transport (ESCRT) machinery, respectively (Luo et al., 2014; Gao et al., 2017). On the other hand, under starvation or other stress conditions, macroautophagy (hereafter simply autophagy), as another major conserved mechanism, mediates turnover and recycling of cytoplasmic materials such as aggregated proteins, damaged or aging organelles (Mehrpour et al., 2010; Liu and Bassham, 2012; Gao et al., 2014; Anding and Baehrecke, 2017). In the autophagic process, cellular contents are engulfed by a double-membrane organelle called the autophagosome (Mizushima et al., 2011; Soto-Burgos et al., 2018), which eventually fuses with the vacuole (Zhuang et al., 2018; Figure 1). Although the MVB and autophagosome pathways have been well studied, the interface between them has rarely been addressed. Interestingly, recent evidence supports a possible crosstalk between these two pathways as some key regulators have been found to localize on both organelles to mediate their interplay (Isono et al., 2010; Katsiarimpa et al., 2011; Katsiarimpa et al., 2013; Kwon et al., 2013; Gao et al., 2015; Nagel et al., 2017).

In this mini review, we summarize the recent advances in research into the MVB and autophagosome pathways in plant cells. The key regulators and their conserved domain will be highlighted. In the end, we also discuss the possible crosstalk between the MVB and autophagosome pathways in plant cells.

## The MVB-Mediated Vacuolar Trafficking Pathway

In the plant secretory pathway, soluble vacuolar cargoes are recognized by VSR proteins (Foresti et al., 2010; Zouhar et al., 2010; Shen et al., 2014; Robinson and Neuhaus, 2016), while in the endocytic pathway, plasma membrane proteins destined for degradation are recognized and internalized into intraluminal vesicles (ILVs) of MVBs via the ESCRT machinery (Valencia et al., 2016; Gao et al., 2017; Isono and Kalinowska, 2017; Figure 1). In the following, we summarize recent findings on the structural features and domain functions of VSR, ESCRT proteins, as well as other plant-unique components in MVB-mediated vacuolar trafficking pathways.

## VSR-Binding and Vacuolar Trafficking of Cargo Proteins

The first VSR protein identified is BP-80 from pea (*Pisum sativum*) (Kirsch et al., 1994). BP-80 recognizes the vacuolar cargo aleurain by binding to an NPIR-containing sequence motif, the most well-studied vacuolar sorting determinant (VSD) of vacuolar-targeting proteins (Paris and Neuhaus, 2002; Watanabe et al., 2004). VSRs are type I integral membrane family proteins with a large N-terminal luminal domain (NT), a single transmembrane domain (TMD), and a short C-terminal cytosolic tail (CT). The luminal region, VSRNT, consists of a protease-associated (PA) domain, a central domain, and an epidermal growth factor (EGF) repeats (Cao et al., 2000; Robinson and Neuhaus, 2016). The TMD and CT domains are responsible for targeting VSRs to the vacuole in plant cells. The CT domain contains a YMPL motif and IM motif (Dasilva et al., 2006; Foresti et al., 2010). The YMPL motif is recognized by the AP-1 clathrin adaptor protein complex and is involved in the formation of clathrin-coated vesicles (CCVs), which is required for MVB targeting while the IM motif is involved in VSR recycling.

The structure of PA domain of VSR from *Arabidopsis thaliana* has been resolved to show how VSRs recognize their cargoes recently (Luo et al., 2014). The crystal structures presented for PA of VSR isoform 1 (VSR1PA) are from *A. thaliana* alone and complexed with a cognate peptide containing the barley (*Hordeum vulgare*) aleurain VSD sequence of _1_ADSNPIRPVT_10_. In this model, the cargo-binding pocket of PA domain is occupied by switch III residues (_130_TPEE_133_), forming a closed conformation before interaction with the cargo. The Ala-Asp-Ser residues preceding the NPIR motif in the cargo (aleurain) are recognized by PA domain and the Arg-95 forms a hydrogen bond to Ser residue, which is crucial to receptor-cargo interaction. When Ala-Asp-Ser residues are bound to the PA domain, they displace the switch III residues from the cargo binding pocket and induce conformational changes that are propagated to the C-terminus of the PA domain. This results in a 180° flip of the C-terminal tail and the conformation is stabilized by hydrogen bond between Glu-24 and His-181, allowing the central domain to cooperate with the PA domain in recognizing the full-length VSD.

## The Role of the ESCRT Machinery in Endosomal-Vacuolar Trafficking

The ESCRT machinery is an assembly of protein subcomplexes which plays canonical roles in the MVB biogenesis and ubiquitinated membrane proteins sorting for degradation and it is evolutionarily conserved in eukaryotes (Leung et al., 2008; Henne et al., 2011). Compared to yeast and animal, plant genome encodes most ESCRT isoforms, including ESCRT-I (VPS23A/VPS23B, VPS28-1/VPS28-2, VPS37-1/VPS37-2), ESCRT-II (VPS22, VPS25, VPS36), ESCRT-III (VPS2-1/VPS2-2/VPS2-3, VPS20-1/VPS20-2, VPS24-1/VPS24-2, SNF7-1/SNF7-2), and VPS4/SKD1 (suppressor of K+ transport growth defect 1) complex with the exception of the canonical ESCRT-0 subunits and ESCRT-I component MVB12 (Otegui and Spitzer, 2008; Richardson et al., 2011; Gao et al., 2017). Here, in comparison with yeast and animals we discuss the canonical functions of the ESCRT machinery in plant endosomal sorting with respect to the structure and functional domains of the subunits. Moreover, we provide some recent findings in plant unique ESCRT components and related proteins (Table 1).

**Table 1:** Key components and regulators in plant MVB and autophagosome pathways discussed in this study.

Complex	Proteins	Activity	Reference(s)
**Plant ESCRT components and regulators**			
Proteins show functional analogies to ESCRT-0	TOL1-TOL9	Binds to ubiquitin, regulates cargo recognition, and interacts with clathrin for ubiquitinated cargo clustering. Contains conserved VHS domains, GAT domains and putative clathrin binding motifs.	Raiborg and Stenmark, 2002; Korbei et al., 2013
ESCRT-I	VPS23A/ELC	Binds to ubiquitin. Forms a putatively intact plant ESCRT-I complex with VPS37 and VPS28.	Spitzer et al., 2006
	VPS23B		
ESCRT-II	VPS36	Binds ubiquitin and forms an ESCRT-II complex with VPS22 and VPS25.	Wang et al., 2017
ESCRT-III accessory proteins	CHMP1A	Interacts with the VPS4/SKD1-LIP5 complex.	Spitzer et al., 2009; Katsiarimpa et al., 2011; Cai et al., 2014
	CHMP1B		
VPS4 accessory proteins	LIP5	Interacts with ISTL1.	Wang et al., 2014, 2015; Buono et al., 2016
ESCRT-related regulators	BRO1/ALIX	Interacts with SNF7, and recruits AMSH3 to the late endosomes to remove ubiquitin from cargoes. Incorporates into the ESCRT-I complex via direct interaction with VPS23A. Recognizes and sorts ubiquitinated cargoes into the ILVs.	Cardona-Lopez et al., 2015; Kalinowska et al., 2015; Shen et al., 2016
	SH3P2	Binds and transfers ubiquitinated proteins to the ESCRT machinery through interaction with VPS23 and AMSH3.	Nagel et al., 2017
	BRAF	Regulates FREE1 recruitment to the MVB membrane by competitively binding VPS23.	Shen et al., 2018
Plant specific ESCRT proteins	FREE1/FYVE1	Binds to PtdIns3P and ubiquitin. Interacts with VPS23 via the PTAP-like tetrapeptide motifs.	Gao et al., 2014; Kolb et al., 2015
	PROS	Regulates VPS4/SKD1 ATPase activity by interacting with LIP5.	Reyes et al., 2014
**Key components in plant autophagy**			
ATG1/ULK1 complex	ATG1a ATG1b ATG1c ATG1t	Phosphates ATG9, recruits ATG8-PE to PAS and initiates autophagy pathway.	Suttangkakul et al., 2011
	ATG13a ATG13b	Interacts with ATG1 and regulates autophagic bodies deposition.	Suttangkakul et al., 2011
	ATG11	Interacts with ATG13 and ATG8-PE and regulates phosphorylation of ATG1.	Li and Vierstra, 2014
ATG9 complex	ATG9	Facilitates formation of ATG9 vesicles and initiates autophagosome progression from the ER membrane.	Zhuang et al., 2017

In yeast, MVB-mediated sorting of ubiquitinated cargoes starts with cargo capture by ESCRT-0, which consists of two subunits Vps27 and Hse1 (Raiborg and Stenmark, 2009). These subunits interact via coiled-coil GAT (GGAs and Tom) domains and recognize cargoes via ubiquitin-interacting motif (UIM) and a VHS (Vps-27, HRS, STAM) domain. ESCRT-0 subunits are absent in plants, however, there exists in the *Arabidopsis* genome nine TOM1-like (TOL) proteins with conserved VHS domains followed by GAT domains and putative clathrin binding motifs. Previous studies have shown that some *Arabidopsis* TOLs can bind to ubiquitin and regulate cargo recognition, as well as interact with clathrin for ubiquitinated cargo clustering (Raiborg and Stenmark, 2002; Korbei et al., 2013).

As displayed by crystal structure, the yeast ESCRT-I complex is an elongated heterotetramer of 20 nm (Kostelansky et al., 2007). The ubiquitin E2 variant (UEV) domain of ESCRT-I Vps23 binds to the PTAP-like motifs of the ESCRT-0 subunit Vps27 (Kostelansky et al., 2006). The *Arabidopsis* homolog VPS23 also has the ability to bind to ubiquitin and to form a putatively intact plant ESCRT-I complex in association with VPS37 and VPS28 (Spitzer et al., 2006).

As demonstrated in yeast, ESCRT-II is a Y-shaped heterotetramer (Langelier et al., 2006). The GLUE (GRAM-like ubiquitin-binding in EAP45) domain of the ESCRT-II subunit Vps36 interacts with the C-terminus of the ESCRT-I subunit Vps28 (Teo et al., 2006). Together with the ESCRT-0 FYVE domain, the GLUE domain provides endosomal localization specificity by binding PtdIns3P. In *Arabidopsis*, recent studies have shown that VPS36 might form an ESCRT-II complex with VPS22 and VPS25 and also shows ubiquitin-binding activity, regulating MVB biogenesis as well as the endosomal sorting of membrane cargoes (Wang et al., 2017).

In yeast, ESCRT-III recruitment to the endosome and complex formation is initiated when the ESCRT-II subunit Vps25 binds to Vps20, which drives membrane invagination and scission of ILVs (Henne et al., 2013). All the isoforms of ESCRT-III subunits are present in *Arabidopsis* and play an essential role in MVB biogenesis, vacuolar sorting as well as embryonic/seedling development. In particular, the *Arabidopsis* Charged multivesicular body protein 1 (CHMP1) proteins have been reported to interact with the VPS4/SKD1 complex to regulate MVB biogenesis and vacuolar sorting of auxin transporters (Spitzer et al., 2009; Katsiarimpa et al., 2011; Cai et al., 2014).

Dissociation of the ESCRT-III complex from the membrane requires energy, and it is provided by the class I AAA (ATPases associated with various cellular activities) ATPase VPS4. The N-terminal microtubule-interacting and trafficking (MIT) domain of VPS4 recognizes and binds to C-terminal MIMs (MIT-interacting motifs) present in the ESCRT-III subunits. LIP5 (lyst-interacting protein 5), a VPS4/SKD1 positive regulator, was recently reported to regulate MVB biogenesis and MVB-mediated sorting of membrane proteins through interaction with increased salt tolerance 1-like1 (ISTL1), a protein predicted to be the *Arabidopsis* homolog of yeast IST1 (increased salt tolerance 1) (Wang et al., 2014, 2015; Buono et al., 2016).

In addition, recent studies have identified some plant unique ESCRT components, like FYVE domain protein required for endosomal sorting 1 (FREE1) and positive regulator of SKD1 (PROS) (Gao et al., 2014; Reyes et al., 2014). ESCRT-related proteins have also been characterized. One example would be *Arabidopsis* BRO1 (or ALIX), which is homologous to yeast bypass of C kinase 1 (BCK1)-like resistance to osmotic shock 1p (Bro1p) and mammalian apoptosis linked gene-2 interacting protein X (ALIX). BRO1/ALIX interacts with the ESCRT-III component SNF7, and recruits the deubiquitinase AMSH3 (associated molecule with the SH3 domain of STAM 3) to late endosomes to remove ubiquitin from cargoes before luminal sequestration of MVBs (Cardona-Lopez et al., 2015; Anding and Baehrecke, 2017). A more recent study demonstrates that BRO1/ALIX is also incorporated into the ESCRT-I complex via direct interaction with VPS23A, recognizing and sorting ubiquitinated cargoes into the ILVs of MVBs for vacuolar degradation (Shen et al., 2016). Another example is the Src homology-3 (SH3) domain-containing protein 2 (SH3P2). It has been shown that SH3P2 is a ubiquitin-binding protein that binds and transfers ubiquitinated proteins to the ESCRT machinery through interaction with ESCRT-I subunit VPS23 and the deubiquitinating enzyme AMSH3 (Nagel et al., 2017). In addition, another latest report has demonstrated that a plant Bro1-domain protein as FREE1 suppressor (BRAF) functions as a unique evolutionary ESCRT regulator (Shen et al., 2018). BRAF regulates FREE1 recruitment to the MVB membrane by competitively binding to the ESCRT-I component VPS23, thus functioning in MVB biogenesis and membrane protein sorting.

## Autophagosome-Mediated Protein Degradation

Autophagy is known to be tightly controlled by the conserved autophagy-related (ATG) proteins (named Atg in yeast and ATG in mammals/plants) (Klionsky et al., 2012). Since the first *ATG* gene was identified in yeast (Tsukada and Ohsumi, 1993), around 40 *ATG* genes have been screened out (Ohsumi, 2014). These core Atg proteins are evolutionarily conserved among eukaryotes and can be classified into five subgroups: the Atg1 complex, the class III phosphoinositide 3-kinase (PI3K) complex, the Atg9 complex, and two ubiquitin-like conjugation systems (Atg5-Atg12 and Atg8) (Mizushima et al., 2011).

To elucidate the mechanism of autophagosome formation, functional and structure biological efforts have been made for decades. However, little structure biological studies have been published on plant ATG components. As recent structure biological progresses on PI3K complex and conjugation systems have been well summarized (Suzuki et al., 2017), in the following we mainly focus on very recent progress about the ATG1 complex and ATG9-ATG2-ATG18 complex (Table 1).

## The ATG1/ULK1 Complex

The budding yeast autophagy Atg1 complex consists of five components (Atg1, 13, 17, 29, and 31) (Kraft et al., 2012; Mao et al., 2013a). The formation of this pentameric complex is induced under nutrient-starvation conditions. However, the scaffolding subcomplex Atg17-Atg31-Atg29 is missing in mammalian (Hurley and Young, 2017) and plant cells (Suttangkakul et al., 2011).

The serine/threonine kinase Atg1 consists of an N-terminal kinase domain (KD), and two C-terminal tandem MIT domains (MIT1 and MIT2), which directly recognize Atg13 (Fujioka et al., 2014). As the sole kinase in autophagy machinery, Atg1 can phosphorylate Atg9, thereby recruiting Atg18 and Atg8-phosphatidylethanolamine (PE) to the phagophore assembly site (PAS) (Papinski et al., 2014). Atg13 comprises a Hop1, Rev7, and Mad2 (HORMA) domain at the N-terminus and an intrinsically disordered region (IDR) (Yamamoto et al., 2016). The flexible conformation of Atg13 determines that it functions for complex assembly and substrate recruitment. The structural basis is that the IDR consists of two Atg17-binding regions and MIM (Yamamoto et al., 2016). These two Atg17-binding regions can bind to two individual Atg17 dimers independently. The MIM domain of Atg13 binds to Atg1 and HORMA domain recruits Atg9 vesicles, respectively. On the other hand, Atg17 can form a S-shaped homodimer by four α-helices and subsequently exposes the concave face toward the Atg29-Atg31 heterodimer (Ragusa et al., 2012).

*ATG1* orthologs in *Arabidopsis* comprise four members (*ATG1*a-c and t), while *ATG13* is encoded by a pair of genes (*ATG13a* and *ATG13b*) (Suttangkakul et al., 2011). The remaining 3 components of the ATG1 complex (ATG17, ATG29, ATG31) are not identified in *Arabidopsis*. Like the yeast ortholog, the *ATG1a-c* and *ATG1t* genes were predicted to encode a N-terminal Ser/Thr protein kinase domain around the 260-residue (Suttangkakul et al., 2011). However, the alignment of the kinase domain in angiosperms phylogenetically distinguishes ATG1t isoforms with ATG1a and ATG1b/c clades. Consequently, ATG1t was proposed to represent a novel adaptation to the ATG1 kinase family in seed plants. The lack of ATG1 or ATG13 did not impair ATG8 lipidation but inhibited the forming of autophagic bodies inside the vacuole, indicating an essential role of ATG1 complex in regulating autophagosome enclosure and/or vacuolar delivery (Suttangkakul et al., 2011).

Autophagy was considered as a non-selective degradation pathway for a long time. However, recent research has revealed that autophagy is also critical for removal of certain cargoes, like damaged or superfluous organelles and protein aggregates (Mao et al., 2013b; Lu et al., 2014; Khaminets et al., 2015; Nakamura et al., 2018). As a scaffold protein, Atg11 is essential in organizing selective autophagy-specific PAS via interacting with various Atg proteins and cargo receptors (Liu and Klionsky, 2016). So far *ATG11* has been identified in yeast (Shintani et al., 2002) and *Arabidopsis* (Li and Vierstra, 2014), while its ortholog is defined as Focal Adhesion Kinase Family-Interacting Protein of 200 kD (FIP200) in mammals (Hara et al., 2008). Since it fails to identify *ATG17* in *Arabidopsis*, ATG11 protein is likely shared by both bulk and selective autophagy processes. Except for the conserved four coiled-coil (CC) motifs and a C-terminal ATG11 domain, a short cryptic ATG17-like domain (residues 348–494) was also identified near the N-terminal of *Arabidopsis* ATG11. Recently Atg11 was purified and subjected to a series of biophysical analyses, including analytical ultracentrifugation and CD spectroscopy (Suzuki and Noda, 2018). It turns out that Atg11 can form elongated, dimeric coiled-coil architecture in solution. And homodimerization in both N-terminal and C-terminal implied that Atg11 has a parallel dimeric architecture, in contrast to the antiparallel dimeric architecture in Atg17 (Suzuki and Noda, 2018). The architectural difference between Atg11 and Atg17 may imply a distinct organization of Atg1 complex binding scaffold proteins during phagophore initiation in selective and non-selective autophagy (Yamamoto et al., 2016). Inspiration may be gained from this study in plant ATG11 structure analysis.

## ATG9 AND ATG2-ATG18 Complex

Atg9 is a transmembrane protein, first identified in budding yeast, which possesses six transmembrane domains (Rao et al., 2016). When inserted into a vesicle with diameter around 30–60 nm, termed Atg9 vesicle (Figure 1), both N- and C-termini are exposed to the cytosol (Yamamoto et al., 2012; Rao et al., 2016; Zhuang et al., 2017). In response to autophagic induction, Atg9 vesicles are recruited to PAS and deliver membrane/lipid for autophagosome formation (Yamamoto et al., 2012). Although the intracellular route of Atg9 trafficking remains unclear, the Golgi-endosomal system has been demonstrated to be the key membrane source to produce Atg9 vesicles in yeast (Yamamoto et al., 2012), mammals (Shirahama-Noda et al., 2013), and plants (Zhuang et al., 2017). The HORMA domain of Atg13 has been demonstrated to be able to recruit Atg9 vesicles to PAS and interconnects with the autophagosomal membrane via fusion (Suzuki et al., 2015). Interestingly, *Arabidopsis* ATG9 vesicles display a transient association with the phagophore membrane (Figure 1; Zhuang et al., 2017). ATG9 deficiency leads to an extensive accumulation of autophagosome-related tubular structures decorated by YFP-ATG8e puncta upon autophagic induction. The production of such tubular structures depends on PI3K activity and the direct connection between the tubular structure and ER membrane has been confirmed by confocal analysis and electron tomography (Zhuang et al., 2017). Combined with the distinct distribution in yeast and mammals, it is tempting to speculate that ATG9 may acquire a plant-specific manner in autophagic degradation pathway.

As demonstrated in yeast, Atg2 is the largest Atg machinery protein. A homology search shows that Atg2 contains five domain/motifs with unknown function. Yeast two-hybrid analysis against Atg9 has showed the region PM3 (Asp^1255^-Asp^1259^) is essential for interaction between Atg2 and Atg9 (Gómez-Sánchez et al., 2018). Atg2 works as a bridge and promotes the contact between autophagosomal membranes and the ER. Interaction between Atg2 and Atg9 could be a likely prerequisite for both a close association with the ER and efficient autophagosome biogenesis. Atg18 belongs to the β-propellers that bind polyphosphoinositides (PROPPIN) family, which is essential for PtdIns3P and PtdIns(3, 5)P2 binding (Busse et al., 2015). The ATG18 homolog in mammals is called WD-repeat protein interacting with phosphoinositides (WIPI), which is a protein family containing 4 candidates (WIPI 1-4), and serves as PtdIns3P effector at PAS (Proikas-Cezanne et al., 2015). A recent study elucidated one member of rat ATG2-WIPI complex, ATG2B-WDR45, possessed a club-shaped architecture by negative staining electron microscopy (Zheng et al., 2017). The conserved H/YF aromatic motif in the C-terminal of ATG2A/ATG2B is necessary for binding to WIPI4/WDR45, but not with other three WIPI proteins. Additionally, a 3D reconstruction on human ATG2A-WIPI4 resolved that ATG2A possesses a rod-shape structure and WIPI4 is flexibly associated with ATG2A (Chowdhury et al., 2018).

## Crosstalk Between the MVB and Autophagosome Pathways

In mammals, before fusing with lysosomes, autophagosomes can undergo a maturation process by interacting with MVBs to form a structure called amphisome, which was demonstrated by electron microscopy study (Seglen et al., 1991; Berg et al., 1998). Recent studies showed that dysfunction of the ESCRT machinery led to autophagic defect in mammalian cells. For example, one study showed that inactivation of the ESCRT-0 component TOM1 led to accumulation of autophagosomes and failed to form autolysosomes (Tumbarello et al., 2012). Another study showed that loss of ESCRT-0 HRS resulted in insufficient autophagic clearance and enhanced ER stress (Oshima et al., 2016). Moreover, the ESCRT-III component CHMP2A was reported to be a critical regulator of phagophore closure (Takahashi et al., 2018). In yeast, autophagosomes are likely to fuse with the vacuole directly and evidence for the amphisome intermediate is missing (Knaevelsrud and Simonsen, 2012; Nascimbeni et al., 2017). However, recent research pointed out the coordinated action between the MVB pathway and autophagy was critical for cell survival during periods of starvation (Muller et al., 2015). This coordinated action included several steps: (1) During the first 3 h of starvation in the yeast cells, many integral PM proteins underwent endocytosis and degradation in vacuoles via MVBs; (2) This degradation maintained critical amino acid levels to allow cells to synthesize new proteins at the early stage of starvation; (3) The de novo synthesis of vacuolar hydrolases enhanced the vacuolar catabolic activity and promoted cellular adaptation. Therefore, the efficient vacuolar degradation of materials via autophagy could be achieved at the late stage; whereas the coordinated action of the MVB pathway and autophagy thus allowed cells to survive during starvation (Muller et al., 2015). In plants, some ESCRT proteins (e.g., FREE1) that are involved in the MVB pathway are also found to play additional roles in the autophagy pathway. In the *free1* mutants, the hybrid structures between autophagosomes and MVBs are also observed by electron microscopy, however, the mechanism underlying their fusion is still unknown (Gao et al., 2014, 2015; Zhuang et al., 2015). Besides, recent studies reveal that the RAB7 GTPases localize on both MVBs and autophagosomes that may potentially participate in their crosstalk (Figure 1; Kwon et al., 2013; Cui et al., 2014; Ebine et al., 2014; Singh et al., 2014). The following is a discussion about the roles of a plant-unique ESCRT component, FREE1 and the RAB7 GTPase in plants.

FREE1 is known to bind to phosphatidylinositol-3-phosphate and ubiquitin then interacts with the ESCRT-I subunit VPS23 via the PTAP-like tetrapeptide motifs or possibly incorporated into ESCRT-III via association with SNF7 on MVBs (Gao et al., 2014; Kolb et al., 2015; Belda-Palazon et al., 2016). Meanwhile, FREE1 has also been found on autophagosomes to interact with SH3P2, which is a key regulator in the autophagic pathway (Zhuang et al., 2013; Gao et al., 2015). The mutation of FREE1 induces the formation of abnormal MVB-autophagosome hybrid structures, further implying a possible crosstalk between these two organelles (Figure 1; Gao et al., 2015). Another player localized on both MVBs and autophagosomes is RAB7. Under normal condition, RAB7 localized on both MVBs and vacuoles and mediated the transport between them (Cui et al., 2014; Ebine et al., 2014; Singh et al., 2014), while under pathogen infection, RABG3b (an *Arabidopsis* RAB7 homolog) colocalized with ATG8a in autophagic structures in immunogold TEM study and positively regulated autophagy and immunity-associated hypersensitive cell death in *Arabidopsis* (Kwon et al., 2013). In addition, recent research in yeast found that, a direct interaction of Atg8 with Ypt7 guanosine exchange factor (GEF), Mon1-Ccz1 via an LIR (LC3-Interacting region) motif in the Ccz1 C-terminus, but this motif is not essential for normal endosomal transport (Gao et al., 2018). However, it is still unknown how Mon1-Ccz1 is temporally and spatially recruited to autophagosomes. In addition to the MVB pathway, the autophagy could potentially interplay with other pathways including endocytosis and exocytosis. For example, a recent study found that, in plant cells after autophagy induction, the vesicles labeled by EXO70B1, one of 23 paralogs of *Arabidopsis* EXO70 exocyst subunits, were internalized into the central vacuole and co-localized with the autophagosomal marker ATG8f (Kulich et al., 2013).

## Conclusion and Future Prospects

Substantial progress has been made in identifying regulators important for the crosstalk between the MVB and autophagosome pathways in mammals and yeast (Filimonenko et al., 2007; Lee et al., 2007; Tumbarello et al., 2012; Muller et al., 2015; Oshima et al., 2016; Takahashi et al., 2018). In plants, the MVB and autophagosome pathways mediate protein transport to the vacuole in normal and stress conditions, respectively. So far only a few groups reported endosomal proteins participating in autophagic pathway (Katsiarimpa et al., 2013; Gao et al., 2015; Spitzer et al., 2015). Many outstanding questions still remain to be answered in future research. For example, can MVBs fuse directly with autophagosomes in plants, and if so, what are the underlying mechanisms in regulating MVB-autophagosome fusion? Does the autophagy coordinate with the MVB pathway under stress or in response to nutrient limitations, and if so, what is the biological significance of their crosstalk? Since some of the conserved ATG components are missing in plants, do the regulators in the MVB pathway substitute their function? Future studies on identification and characterization of new regulators involved in both the MVB and autophagosome pathways would lead us to a better understanding of their crosstalk in plants.

## Author Contributions

YC, YH, WC, JG, and LJ designed the concept and the organized the manuscript. YC, YH, and WC wrote the manuscript. YC and LJ edited the manuscript.

## Conflict of Interest Statement

The authors declare that the research was conducted in the absence of any commercial or financial relationships that could be construed as a potential conflict of interest.
